# Clinical characteristics, treatment patterns, and survival outcomes in ductal prostate cancer: a multicenter retrospective analysis

**DOI:** 10.1007/s00345-026-06686-6

**Published:** 2026-08-22

**Authors:** A. Handke, P. Paffenholz, K. Schlack, K. E. Seifert, J. Linxweiler, J. Mink, F. Flockerzi, T. Nestler, M. Wenzel, C. Siech, C. Darr, V. Grünwald, F. Roghmann, K. H. Tully

**Affiliations:** 1 Department of Urology, Marien Hospital Herne, University Hospital Bochum, Bochum, Germany; 2 Department of Urology, University Hospital Cologne, Cologne, Germany; 3 Department of Urology, University Hospital Münster, Münster, Germany; 4 Department of Urology, Saarland University, Homburg, Germany; 5 Department of Pathology, Saarland University, Homburg, Germany; 6 Department of Urology, Bundeswehr Central Hospital, Koblenz, Germany; 7 Department of Urology, Goethe University Hospital Frankfurt, Frankfurt am Main, Germany; 8 Department of Urology, Department for Medical Oncology, University Hospital Essen, Essen, Germany; 9 Interdisciplinary Genitourinary Oncology, Clinic for Urology, Clinic for Medical Oncology, University Hospital Essen, Essen, Germany; 10 Department of Urology and Neurourology, Marien Hospital Herne, Ruhr-University Bochum, Hölkeskampring 40, 44625 Herne, Germany

**Keywords:** Prostate cancer, Ductal prostatic adenocarcinoma, Disease progression, Systemic therapy free survival, Histologic variant

## Abstract

**Purpose:**

Prostatic ductal adenocarcinoma (PDA) is a rare, aggressive prostate cancer subtype associated with advanced disease and poorer prognosis than acinar adenocarcinoma. Small ductal components influence management and preclude active surveillance. This study analyzed oncologic outcomes and therapeutic patterns in a multicenter German cohort.

**Methods:**

Retrospective multicenter cohort, seven high-volume German centers. 82 patients with histologically confirmed PDA diagnosed 2014–2024 included. Histopathological data from biopsy and radical prostatectomy (RP) specimens. Clinical data: local/systemic treatments, follow-up until 2025. Primary outcome: time to systemic therapy. Secondary: overall survival. Predictors of adverse outcomes assessed via Cox regression.

**Results:**

Most patients presented with d’Amico high-risk (47/82, 57.3%) or intermediate-risk (28/82, 34.2%) disease; 9.8% (8/82) had synchronous metastases. RP performed in 82.9% (68/82), radiotherapy in 7.3% (6/82). Postoperative lymph node positivity and positive surgical margins occurred in 25.0% (17/68) and 22.1% (15/68), respectively. Median follow-up was 36 months (IQR 20–54 months). At the time of analysis, overall survival and cancer-specific survival were immature, with a limited number of events during follow-up. Among patients who required systemic therapy during follow-up, the median interval from local treatment to initiation of systemic therapy was 23 months (IQR 5–31 months). High pre-/postoperative Gleason scores (≥ 8) were associated with earlier initiation of systemic therapy. Among patients receiving systemic treatment (*n* = 24), ADT monotherapy showed longer median duration (27.2 months) than ARPI (10.9 months) or chemotherapy (6.1 months). Limitations: retrospective design, limited sample size.

**Conclusion:**

PDA demonstrates aggressive behavior with early need for systemic therapeutic escalation in a substantial proportion. High Gleason scores were associated with earlier initiation of systemic therapy. These findings suggest that PDA should be considered a high-risk entity. However, survival outcomes require longer follow-up for definitive conclusions.

## Introduction

Prostate cancer (PCa) remains one of the most prevalent malignancies among men worldwide and a leading cause of cancer-related mortality. While most prostate cancers are acinar adenocarcinomas, prostatic ductal adenocarcinoma (PDA) represents a distinct and aggressive histologic subtype. It is important to distinguish PDA from intraductal carcinoma, a pre-invasive lesion often co-existing with high-grade acinar carcinoma, and from aggressive-variant prostate cancer, characterized by rapid progression and resistance to androgen receptor–targeted therapies. Understanding these less common histologic patterns has evolved in recent years, and as PDA is associated with poor prognosis compared with PAA, accurate recognition of its hallmarks is paramount for clinical decision-making [1]. Ductal histologic patterns are acknowledged in contemporary pathological and clinical consensus recommendations due to their association with aggressive disease characteristics and unfavorable oncologic outcomes. Consequently, the presence of ductal differentiation is generally considered a contraindication to active surveillance strategies in routine clinical practice [2].

In routine histopathological assessment, International Society of Urological Pathology (ISUP) Grade Group upgrading and pathological upstaging are frequently observed at final radical prostatectomy (RP). However, dedicated data on PDA are limited [3]. Clinically, PDA presents at more advanced stages and is associated with higher ISUP GG, increased extraprostatic extension, seminal vesicle invasion, and positive surgical margins following RP [4, 5]. Notably, PDA may exhibit lower serum prostate-specific antigen (PSA) levels despite aggressive nature, potentially leading to underdiagnosis [6]. Overall survival (OS) for metastatic PDA is 72% at 5 years with a median of 77 months [7, 8], and PDA is linked to higher disease-specific mortality and metastasis to atypical sites such as lungs, brain, and testis [7–9]. Even a small ductal component on initial biopsy associates with higher risk of biochemical recurrence (BCR) and early metastatic progression [5, 10].

Given its distinct features, accurate PDA identification is crucial for guiding treatment decisions. This study aims to analyse oncologic outcomes, particularly therapeutic courses, including respective systemic and local treatments for PDA.

## Materials and methods

### Study design

We conducted a retrospective multicenter cohort study including patients diagnosed with prostatic ductal adenocarcinoma across seven high-volume urological centers in Germany between 2014 and 2024. Clinical data were collected and analyzed collaboratively across the participating institutions, following standardized protocols.

### Patient selection

A total of 82 patients with histologically confirmed PDA from seven high-volume urological centers in Germany were included. Only patients with histologically confirmed prostatic ductal adenocarcinoma were included, whereas cases with isolated intraductal carcinoma without ductal adenocarcinoma morphology were not considered part of the study cohort. Diagnosis was established on RP specimens and, where applicable, on prostate biopsy. If available, the percentage of the ductal component was also recorded. Patients with insufficient histopathologic characterization were excluded. The analyzed therapeutic data comprised all treatments after local therapy, biochemical recurrence, and survival until 2025.

### Clinicopathological data collection

Baseline clinicopathological parameters were extracted from institutional databases. They included age at diagnosis, relevant secondary diagnoses, smoking status, preoperative PSA level, clinical T stage, biopsy ISUP grade, and risk classification according to the European Association of Urology (EAU) guidelines. For patients undergoing RP, pathological findings included tumor stage (pT), lymph node status (pN), surgical margin status, and ISUP from the final specimen. Whenever available, the percentage of ductal growth infiltration was assessed. Baseline staging modalities were performed according to institutional standards and evolving guideline recommendations during the study period and included conventional imaging (computed tomography and bone scintigraphy) and, in later years, PSMA-PET/CT where available. Data regarding adjuvant, salvage, or later therapies (androgen deprivation therapy (ADT), androgen receptor pathway inhibitors (ARPI), chemotherapy, or radiotherapy) were recorded.

### Outcome measures

The primary endpoint was time to initiation of systemic therapy following local treatment, defined as the interval between RP or radiotherapy and the initiation of any systemic therapy after the diagnosis of metastatic disease or biochemical recurrence (ADT, ARPI, or chemotherapy).Due to the retrospective multicenter design and the heterogeneous availability and granularity of longitudinal PSA follow-up data across participating institutions, standardized PSA-based biochemical recurrence definitions could not be consistently reconstructed for the entire cohort. Therefore, initiation of systemic therapy was selected as a pragmatic clinically meaningful endpoint reflecting disease progression requiring therapeutic escalation in routine clinical practice. This endpoint included initiation of systemic therapy after biochemical recurrence, or in the context of metastatic disease progression, depending on the documented clinical indication at the treating institution. To avoid including patients undergoing immediate adjuvant radiotherapy or with PSA persistence, only patients receiving systemic therapy at least 6 months after local therapy were included in this analysis.

Secondary endpoints included overall survival (OS) and cancer-specific survival (CSS). Given the limited follow-up duration, survival outcomes were considered exploratory in nature. Additionally, due to the heterogeneity in follow-up documentation, non-curative biochemical recurrence was operationally defined as the initiation of systemic ADT, with or without additional therapy such as ARPI or chemotherapy, but without concomitant local radiotherapy.

### Statistical analysis

Medians and interquartile ranges (IQRs) were reported for continuous variables; frequencies and proportions for categorical variables. Chi-square tests compared categorical variables (e.g., PDA at diagnosis vs. final pathology). Due to limited sample size and events, separate univariable Cox models identified predictors of earlier initiation of systemic therapy, including age, PSA, ISUP (biopsy and post-RP), and pathological T-stage. Results are presented as hazard ratios (HR) with 95% confidence intervals (CI). Kaplan-Meier curves were used to descriptively visualize time to systemic therapy initiation; time zero was diagnosis. Patients without initiation of systemic therapy at last follow-up were censored at the date of last clinical contact or death. Unpaired t-tests compared mean therapy duration. All analyses were conducted using Stata (Version Stata/SE 15.1, Stata Corp LLC, TX, USA). A two-sided p-value < 0.05 was considered statistically significant.

Given the limited number of events and overall sample size, all regression analyses should be considered exploratory and hypothesis-generating in nature.

## Results

### Patient baseline characteristics

A total of 82 patients with histologically confirmed PDA were included across seven high-volume urological centers in Germany. The median age at diagnosis was 69 years (IQR 65–73 years), and the median PSA at the time of diagnosis was 9.7 ng/mL (IQR 6.3–13.8 ng/mL). At the time of diagnosis, the majority of patients presented with high-risk (47/82, 57.3%) or intermediate-risk (28/82, 34.2%) disease. Preoperative staging revealed that eight patients (8/82, 9.8%) had synchronous metastatic disease at the time of diagnosis. In patients with synchronous metastatic disease, the median PSA was 17.3 ng/mL (IQR 8.55–199.9 ng/mL), with five patients showing PSA levels below 20 ng/mL. Further baseline characteristics are listed in Table 1.

**Table 1:** Patient characteristics: baseline characteristics of patients diagnosed with prostatic ductal adenocarcinoma (PDA)Patient and tumor-specific characteristics at the time of biopsy (*n* = 82)

a) Patient and tumor-specific characteristics at the time of biopsy (n=82)		
Median age, years (IQR*)		69 (65–73)
Median PSA^#^ at diagnosis, ng/ml (IQR)		9.7 (6.3–13.8)
Median prostate volume, cm^3^ (IQR)		48 (40–64)
Clinical T-stage (DRE^+^), n(%)	cT1c	36 (43.9)
	cT2a	12 (14.6)
	cT2b	11 (13.4)
	cT2c	8 (9.8)
	cT3	8 (9.8)
	Unknown	7 (8.5)
ISUP at biopsy, n (%)	1	11 (13.4)
	2a	16 (19.5)
	3	22 (26.8)
	4 (Gleason 8)	21 (25.6)
	4 (Gleason 9)	11 (13.4)
	10	1 (1.2)
D’Amico risk classification, n (%)	Low risk	7 (8.5)
	Intermediate risk	28 (34.2)
	High risk	47 (57.3)
Evidence of PDA, n (%)	Yes	40 (48.8)
	No	42 (51.2)
Consecutive therapy, n (%)	Surgery	68 (82.9)
	Radiotherapy	6 (7.3)
	Systemic therapy (primary metastatic disease)	8 (9.8)
b) Tumor-specific characteristics of patients with PDA undergoing surgery (*n* = 68)		
ISUP at final pathology, n (%)	1	3 (4.4)
	2	16 (23.5)
	3	30 (44.1)
	4 (Gleason 8)	6 (8.8)
	4 Gleaswon 9)	12 (17.7)
	10	1 (1.5)
T-stage, n (%)	pT2b	10 (14.7)
	pT2c	23 (33.8)
	pT3a	16 (23.5)
	pT3b	19 (27.9)
N-stage, n (%)	pN0	51 (75.0)
	pN+	17 (25.0)
Resection status, n (%)	R0	53 (77.9)
	R+	15 (22.1)
Proportion of PDA on final histopathology, n(%)	≤ 5%	27 (39.7)
	6% − 49%	20 (29.4)
	≥ 50%	21 (30.9)

*IQR = Interquartile range; ^#^PSA = prostate specific antigen; ^+^DRE = digital-rectal examination

### Local treatment and pathologic findings

Among non-metastatic patients (*n* = 74, 90.2%), RP was the predominant local therapy (68/74, 91.9%), while 6 patients (6/74, 8.1%) underwent definitive radiotherapy. Postoperative pathological analysis revealed lymph node-positive disease in 18 patients (18/68, 26.5%) and positive surgical margins in 15 patients (15/68, 22.1%). Most patients were diagnosed with ISUP 3 (30/68, 44.1%) or 4 (19/68, 27.9%). Extraprostatic extension and seminal vesicle invasion were observed in 16 (16/68, 23.5%) and 19 (19/68, 27.9%) patients, respectively.

At the time of prostate biopsy, 40 patients (40/82, 48.8%) had PDA; 30 (30/40, 75.0%) of whom underwent surgery. Final histopathology after RP, however, showed an additional 38 cases (38/68, 55.9%) of PDA that had been previously missed by biopsy. There were no patients with PDA at the time of biopsy, but without PDA at the time of RP. In the current cohort, only 7 patients (8.5%) were classified as low-risk. In this subgroup, PDA could be identified by biopsy in 4 patients (57.1%). All low-risk patients underwent local therapy (RP: *n* = 6/7, 85.7%; Radiotherapy: *n* = 1/7, 14.7%). At the time of RP, 3 additional cases without PDA at the time of biopsy could be found.

### Oncologic outcomes

**Fig. 1:**
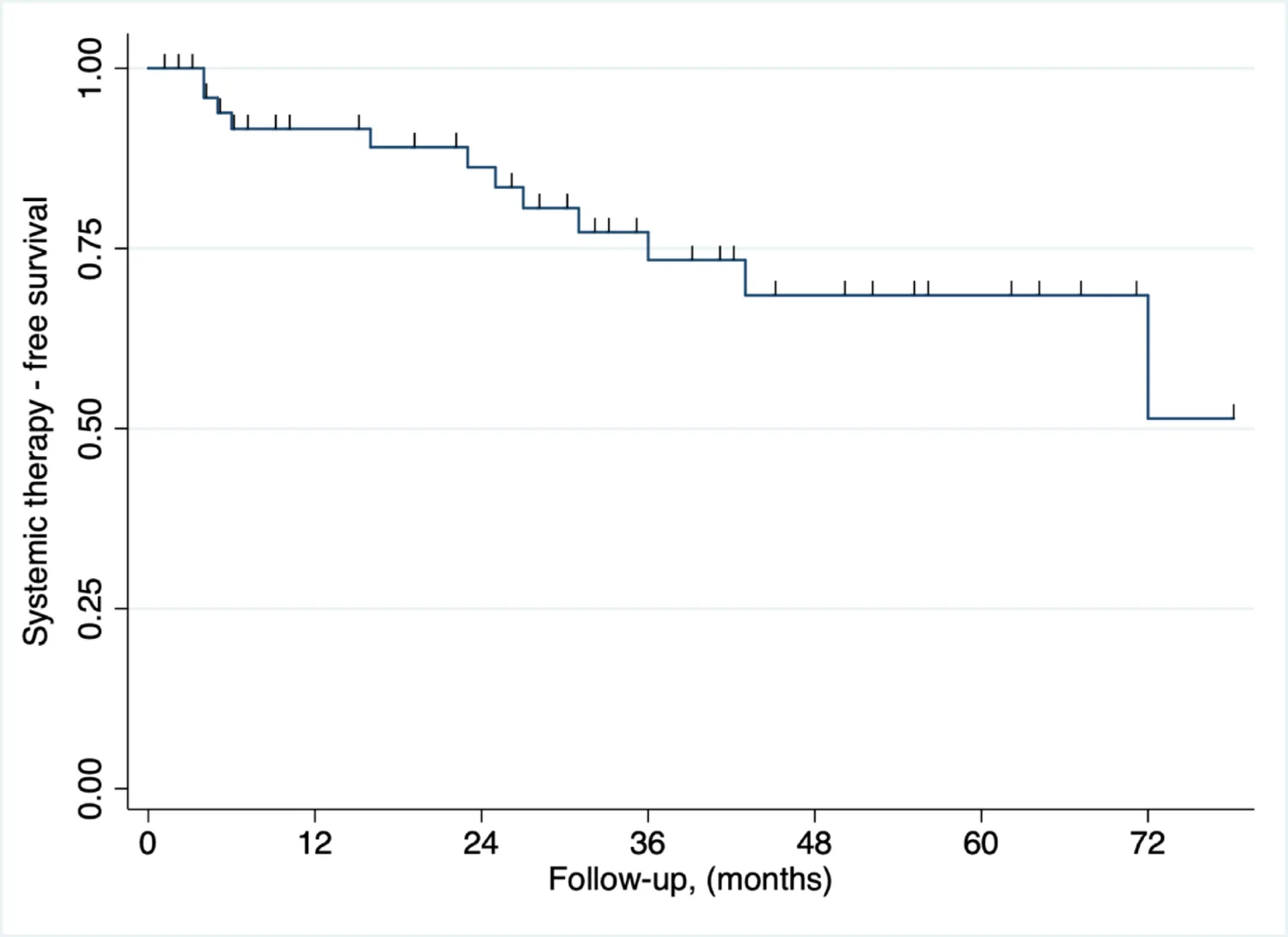
Kaplan–Meier analysis examining the systemic therapy-free survival of patients diagnosed with prostatic ductal adenocarcinoma

Median follow-up for the entire cohort was 36 months (IQR 20–54 months). Including censoring of patients alive at last follow-up, at the 10-year landmark, median OS and CSS were not reached in the overall cohort. Seven patients (7/82, 8.5%) died during the observation period, and 24 (24/82, 29.3%) experienced BCR. Of these 24 patients, 21 (21/24, 87.5%) had previously undergone surgery. Among previously non-metastatic patients who ultimately required systemic therapy during follow-up, the median interval from local treatment to initiation of systemic therapy was 23 months (IQR 5–31 months) (Fig. 1).

### Predictors of unfavorable outcomes

In separate univariable Cox regression analyses, both preoperative and postoperative ISUP ≥ 4 were significantly associated with an increased risk of systemic therapy initiation. Specifically, a preoperative ISUP ≥ 4 showed a hazard ratio (HR) of 3.36 (95%CI1.24–9.14, *p* = 0.017), while a postoperative ISUP ≥ 4 demonstrated an HR of 5.06 (95%CI1.79–14.2, *p* = 0.002). No significant associations were observed for age, PSA at diagnosis, or pathological lymph node status (Table 2).

**Table 2:** Cox proportional hazards regression analyses examining predictors of systemic therapy-free survival

		Hazard ratio	95% Confidence interval	*p*-value
a) Overall cohort				
T-stage	pT2	REF	–	–
	pT3a	0.26	0.30–2.28	0.225
	pT3b	1.91	0.61–5.99	0.265
N-stage	pN0	REF	–	–
	pN+	3.66	1.18–11.4	0.025
ISUP at biopsy	≤ 3	REF	–	–
	≥ 4	3.22	1.27–8.20	0.014
ISUP at final pathology	≤ 3	REF	–	–
	≥ 4	4.15	1.50–11.4	0.006
PSA at diagnosis	≤ 20	REF	–	–
	> 20	2.01	0.77–5.25	0.152
Proportion of PDA on final histopathology	≤ 5%	REF	–	–
	6% − 49%	0.82	0.15–4.54	0.816
	≥ 50%	2.82	0.92–8.65	0.070
b) Non-metastatic patients undergoing surgery				
T-stage	pT2	REF	–	–
	pT3a	0.33	0.03–3.24	0.344
	pT3b	2.42	0.63–9.26	0.198
N-stage	pN0	REF	–	–
	pN+	3.92	1.19–12.9	0.024
ISUP at biopsy	≤ 3	REF	–	–
	≥ 4	2.97	0.87–10.2	0.084
ISUP at final pathology	≤ 3	REF	–	–
	≥ 4	2.96	0.94–9.37	0.065
PSA at diagnosis	≤ 20	REF	–	–
	> 20	1.93	0.51–7.30	0.331
Proportion of PDA on final histopathology	≤ 5%	REF	–	–
	6% − 49%	0.84	0.15–4.71	0.846
	≥ 50%	1.78	0.50–6.30	0.374

### Systemic therapy and response

Overall, 24 patients (24/82, 29.3%) received systemic therapy during follow-up. Additionally, 4 patients previously received ADT within 6 months after local therapy for immediate adjuvant or early salvage radiotherapy. In the first-line, non-curative setting, 14 patients received ADT monotherapy, while 7 patients underwent additional ARPI therapy. 3 patients underwent first-line chemotherapy with docetaxel. Median time to second-line therapy differed by therapeutic modality: 27.2 months (95%CI12–41.6 months) for ADT monotherapy, 10.9 months (95%CI5–16.6 months) for ADT/ARPI-therapy (*p* = 0.085), and 6.1 months (95%CI1.5–10.8 months) for ADT/chemotherapy (*p* = 0.033).

In the subgroup of patients with low-risk disease, 28.7% (*n* = 2/7) underwent further systemic therapy, one after 41 months, the other after 188 months.

## Discussion

In this multicenter retrospective cohort of patients with PDA, our findings are consistent with the aggressive clinical behavior previously attributed to this histologic subtype.

In our cohort, the median PSA at diagnosis was 9.7 ng/mL, reflecting PSA heterogeneity in PDA. This aligns with studies disputing comparable PSA levels between ductal and acinar tumors [11–13]. Packiam et al. showed PSA in localized pure PDA is higher than ISUP 1–2 acinar cancers, yet lower than ISUP 4–5 disease, suggesting PSA reflects tumor differentiation rather than histology alone [13]. That study included only pure ductal carcinoma; our cohort included mixed variants, explaining broader PSA distribution. Mixed histology may lead to higher PSA levels due to a larger acinar component, thereby masking the typically modest PSA secretion associated with pure ductal growth patterns. PSA > 20 ng/mL did not predict earlier systemic therapy (HR 2.01, 95% CI 0.77–5.25), supporting limited prognostic value. This hypothesis is supported by PSA levels in synchronous metastatic patients, with five of eight patients showing PSA levels below 20ng/mL despite metastatic disease. This may contribute to delayed diagnosis and underestimation of disease extent, emphasizing the need for imaging-based staging and careful histopathologic assessment rather than PSA-driven risk stratification.

In our cohort, nearly 10% of patients presented with synchronous metastatic disease at diagnosis, reflecting the well-described propensity of PDA to manifest at an advanced stage. This rate is higher than in typical acinar adenocarcinoma and aligns with reports of early extraprostatic spread. Also, the rate of metastatic disease at diagnosis aligns with the literature, which reports rates ranging from 10% to over 25%, especially in pure ductal cases [14, 15].

Similar to Ranasinghe et al., our cohort showed high BCR (29.3%) and short median time to systemic therapy (23 months), emphasizing aggressive PDA biology [5, 16]. However, the relatively short interval to systemic therapy initiation must also be interpreted in the context of evolving imaging standards during the study period. As PSMA-PET/CT was not uniformly available across all participating centers and years of inclusion, occult micrometastatic disease at baseline may have remained undetected in a subset of patients initially classified as non-metastatic. This potential understaging may have contributed to the early need for systemic treatment observed in our cohort. These findings support that PDA biology is prone to early dissemination, driven by extraprostatic extension or occult micrometastases. Notably, apart from ISUP ≥ 4, neither PSA level nor other conventional clinicopathological parameters reliably predicted the need for systemic therapy, highlighting the limitations of standard risk-stratification tools in this histologic subtype [18]. Early systemic treatment raises the question of earlier multimodal approaches, similar to high-risk PCA. Ranasinghe et al. reported poorer ADT responses in PDA, but our cohort demonstrated measurable ADT activity, suggesting variable endocrine sensitivity [5].

Guidelines and APCCC 2022 suggest aggressive histologic variants may benefit from earlier ADT, potentially neoadjuvant or perioperative [17]. From a clinical perspective, our findings support the consideration of PDA as a clinically high-risk entity, regardless of the percentage of ductal histology, and our findings are consistent with current pathological consensus recommendations that generally discourage active surveillance in the presence of ductal differentiation. Given the tendency toward early systemic progression, upfront consideration of treatment intensification appears biologically plausible. However, PDA-specific trials examining multimodal strategies are clearly needed. Such an approach would require prospective evaluation. Overall, these findings underscore the need for PDA-specific prognostic markers and tailored therapeutic strategies that extend beyond conventional PSA-driven risk stratification.

Notably, ADT monotherapy had longer median duration than ARPI or chemotherapy. At first glance, this may suggest that a subset of PDA retains clinically relevant androgen sensitivity. However, this finding must be interpreted in the context of the disease state and biological selection. Patients historically received ADT monotherapy in routine practice, typically in the hormone-sensitive prostate cancer (HSPC) setting, where androgen receptor (AR) signaling remains the predominant driver of tumor growth and responses to ADT are common [18]. In our cohort, most first-line non-curative patients received ADT (*n* = 14), fewer ARPI (*n* = 7), few chemotherapy (*n* = 3).

ARPI and chemotherapy were used in later stages (CRPC), likely explaining shorter durations rather than inferior efficacy [19]. Stepwise escalation likely explains shorter ARPI/chemotherapy duration versus ADT, not inferior activity. Because dedicated evidence for PDA is limited, therapeutic strategies are frequently extrapolated from PAC. In PAC, adjuvant or salvage ADT, with or without RTx, has been shown to improve disease-free survival in patients with adverse pathological features, including nodal involvement or postoperative recurrence [20]. Consequently, ADT-based approaches have also been widely adopted for PDA, supported by consistent AR expression in most PDA tumors and reports of long-term ADT responses in small RTx cohorts and metastatic series [6, 21–26]. However, PDA does not mirror PAC biology uniformly. Prior work has shown that intrinsic pathway upregulation in PDA can attenuate ADT efficacy, indicating early resistance mechanisms in a subset of tumors [5]. In this context, our finding of longer ADT duration likely reflects the subgroup of PDA that retains AR-driven biology.

In contrast, the shorter treatment windows observed with ARPIs and chemotherapy plausibly represent patients whose disease had already transitioned toward AR-independent or more aggressive phenotypes. These observations are consistent with the concept that PDA comprises both androgen-sensitive and androgen-resistant biological subtypes. Second, the observed differences in treatment duration may also reflect a selection bias, as patients who could remain on ADT alone might have had lower disease burden or slower biological progression, as ADT monotherapy was an established therapeutic concept before dual therapies. Conversely, those escalated early to ARPIs or chemotherapy often represent inherently aggressive disease phenotypes or have already transitioned to CRPC.

Another important finding in our cohort is the considerable discrepancy between diagnostic prostate biopsy and final prostatectomy histology: a substantial proportion of PDA cases were not identified on initial biopsy but only on the surgical specimen. A small subset of patients classified as low-risk according to biopsy-based d’Amico classification showed ductal differentiation only in the radical prostatectomy specimen. This has important clinical implications. If the probability of ductal differentiation is similar across treatment groups in PAC, many patients managed non-surgically are unlikely ever to receive the complete histological assessment that would reveal a ductal component. Consequently, prognostic information may remain unknown, and PDA prevalence underestimated. As contemporary consensus statements increasingly support radiotherapy-based approaches for specific high-risk presentations, the shift toward non-surgical management may amplify this “hidden” burden of PDA [17, 27]. In this context, primary surgical management may offer a unique diagnostic advantage by enabling comprehensive histopathological assessment, which in turn can directly influence postoperative risk stratification and adjuvant treatment decisions. Patients with ductal differentiation might therefore particularly benefit from an initial surgical approach, as radiotherapy-based strategies may preclude definitive histological characterization and thereby limit access to histology-driven therapeutic escalation. In practice, this argues for heightened pathological scrutiny of biopsy specimens, with targeted sampling where feasible, and for careful consideration of surgical management when ductal differentiation is suspected preoperatively.

Furthermore, routine reporting of any ductal features, even if focal, should be established and incorporated into the guidelines. It should be considered if patients with high-risk features despite a negative biopsy for ductal patterns should receive an intensive staging and closer follow-up. Together, these measures could reduce the risk that ductal histology remains occult and ensure that patients receive risk-adapted counseling and treatment planning.

Several limitations of our study must be acknowledged. First, as a retrospective multicenter analysis, treatment selection bias and heterogeneity in management approaches are unavoidable. Moreover, the primary endpoint of systemic therapy-free survival represents a pragmatic clinical endpoint rather than a standardized oncologic surrogate such as PSA-based biochemical recurrence-free survival or metastasis-free survival. As initiation of systemic therapy varied across institutions and clinical contexts, the analyzed endpoint encompassed heterogeneous treatment situations, including biochemical recurrence and initiation of treatment for overt metastatic disease. However, due to incomplete and non-standardized longitudinal PSA follow-up data across centers, established PSA-based recurrence definitions could not be consistently applied in this retrospective cohort. We were therefore unable to establish uniform criteria for biochemical recurrence or harmonize the clinical triggers for initiating systemic therapy across participating centers. In addition, detailed clinical information regarding the specific indication for systemic therapy initiation (adjuvant, salvage, or metastatic setting), as well as comprehensive data on metastatic burden, anatomical distribution, and precise timing relative to disease recurrence, were not consistently available across all participating centers due to the retrospective and multicenter nature of the study. To avoid including patients undergoing immediate adjuvant radiotherapy or early salvage therapy for PSA persistence, only patients undergoing treatment 6 months after initial local therapy were included in the final analysis. Second, the number of patients receiving systemic therapy and the lack of data granularity in this setting limit our analyses to univariable models. Despite the multicenter design, the cohort remains small, highlighting the rarity of this subtype. Given the limited follow-up duration, survival outcomes should therefore be interpreted as exploratory and hypothesis-generating rather than definitive. Second, detailed data on the percentage of ductal growth infiltration at the time of biopsy were unavailable in the majority of cases, limiting biological correlative analyses. Furthermore, baseline staging was not standardized across centers and evolved substantially during the inclusion period, particularly as the implementation of PSMA-PET/CT increased. Consequently, some patients classified as non-metastatic at diagnosis may have harbored occult metastatic disease not detectable by conventional imaging. Detailed pathological characterization of positive surgical margins, including anatomical location and margin extent, was not consistently available across participating centers and therefore could not be systematically analyzed. These features may provide additional prognostic information in PDA and should be addressed in future prospective studies. Finally, follow-up remains relatively short for a disease with a potentially long natural history, and specific outcome measures (e.g., cancer-specific survival stratified by systemic therapy subtype) could not be assessed in a standardized way. Future studies incorporating extended follow-up as well as molecular and genomic characterization may further refine the biological understanding and therapeutic stratification of PDA.

## Conclusion

In patients with PDA, the longer duration of ADT monotherapy compared with ARPI or chemotherapy likely reflects the disease state (HSPC) at the time of therapy initiation rather than inherent treatment superiority. The shorter durations associated with ARPI/chemotherapy may reflect the shift to CRPC, more aggressive tumor biology, and prior selection of high-risk patients. The distinct biology of PDA therefore justifies future prospective studies incorporating early systemic treatment intensification and molecular characterization to better refine therapeutic sequencing and improve patient outcomes. However, given the rarity of PDA, randomized controlled trials are unlikely to be feasible. As a pragmatic alternative, large multicenter retrospective cohort studies with standardized data collection and advanced statistical methodologies, such as propensity score matching across extended (ideally international) datasets, may represent a realistic next step toward generating higher-level evidence for optimal treatment strategies in this patient population.

Given the limited cohort size and follow-up duration, survival and regression analyses should be interpreted as exploratory and hypothesis-generating rather than providing definitive prognostic or clinically actionable conclusions.

## Data Availability

The data that support the findings of this study are available from the corresponding author upon reasonable request.
